# Supplementary material for: End Sequence Analysis Toolkit (ESAT) expands the extractable information from single-cell RNA-seq data
Source: Genome Res. 2016 Oct;26(10):1397–410. doi: 10.1101/gr.207902.116 (PMC5052061; doi:10.1101/gr.207902.116)
Supplement: Supplemental Material [file supp_gr.207902.116_Supp_Materials.tar.gz › ESAT-esat_v0.1/doc/html/interfacebroad_1_1core_1_1primer3_1_1_primer3_picking_summary.html]

ESAT: broad.core.primer3.Primer3PickingSummary Interface Reference


ESAT


- Main Page
- Related Pages
- Packages
- Classes
- Files

- Class List
- Class Index
- Class Hierarchy
- Class Members

All Classes Namespaces Files Functions Variables Enumerator Pages

- broad
- core
- primer3
- Primer3PickingSummary

Public Member Functions |
List of all members

broad.core.primer3.Primer3PickingSummary Interface Reference

|  |  |
| --- | --- |
| Public Member Functions | |
| int | getNumOligosTested () |
|  | |
| int | getNumNs () |
|  | |
| int | getNumOverlappingTargets () |
|  | |
| int | getNumOverlappingExcludedRegions () |
|  | |
| int | getNumGC () |
|  | |
| int | getNumGCClampFailures () |
|  | |
| int | getNumColdMeltingTemp () |
|  | |
| int | getNumHotMeltingTemp () |
|  | |
| int | getNumSelfComplementarityAnywhere () |
|  | |
| int | getNum3PrimeComplementarity () |
|  | |
| int | getNumMispriming () |
|  | |
| int | getNumPolyX () |
|  | |
| int | getNumSeqQuality () |
|  | |
| int | getNum3PrimerStability () |
|  | |
| int | getNumOk () |
|  | |

## Detailed Description

A Primer3PickingSummary is a summary of how many primers primer3 picked in a primer picking session. It includes detailed summary information about why primers were rejected   
  
It's really a java wrapper around the primer3 oligo\_struts typedef struct.

Author
:   Andrew R. Zimmer

## Member Function Documentation

|  |  |  |  |  |
| --- | --- | --- | --- | --- |
| int broad.core.primer3.Primer3PickingSummary.getNum3PrimeComplementarity | ( |  | ) |  |

|  |  |  |  |  |
| --- | --- | --- | --- | --- |
| int broad.core.primer3.Primer3PickingSummary.getNum3PrimerStability | ( |  | ) |  |

|  |  |  |  |  |
| --- | --- | --- | --- | --- |
| int broad.core.primer3.Primer3PickingSummary.getNumColdMeltingTemp | ( |  | ) |  |

|  |  |  |  |  |
| --- | --- | --- | --- | --- |
| int broad.core.primer3.Primer3PickingSummary.getNumGC | ( |  | ) |  |

|  |  |  |  |  |
| --- | --- | --- | --- | --- |
| int broad.core.primer3.Primer3PickingSummary.getNumGCClampFailures | ( |  | ) |  |

|  |  |  |  |  |
| --- | --- | --- | --- | --- |
| int broad.core.primer3.Primer3PickingSummary.getNumHotMeltingTemp | ( |  | ) |  |

|  |  |  |  |  |
| --- | --- | --- | --- | --- |
| int broad.core.primer3.Primer3PickingSummary.getNumMispriming | ( |  | ) |  |

|  |  |  |  |  |
| --- | --- | --- | --- | --- |
| int broad.core.primer3.Primer3PickingSummary.getNumNs | ( |  | ) |  |

|  |  |  |  |  |
| --- | --- | --- | --- | --- |
| int broad.core.primer3.Primer3PickingSummary.getNumOk | ( |  | ) |  |

|  |  |  |  |  |
| --- | --- | --- | --- | --- |
| int broad.core.primer3.Primer3PickingSummary.getNumOligosTested | ( |  | ) |  |

|  |  |  |  |  |
| --- | --- | --- | --- | --- |
| int broad.core.primer3.Primer3PickingSummary.getNumOverlappingExcludedRegions | ( |  | ) |  |

|  |  |  |  |  |
| --- | --- | --- | --- | --- |
| int broad.core.primer3.Primer3PickingSummary.getNumOverlappingTargets | ( |  | ) |  |

|  |  |  |  |  |
| --- | --- | --- | --- | --- |
| int broad.core.primer3.Primer3PickingSummary.getNumPolyX | ( |  | ) |  |

|  |  |  |  |  |
| --- | --- | --- | --- | --- |
| int broad.core.primer3.Primer3PickingSummary.getNumSelfComplementarityAnywhere | ( |  | ) |  |

|  |  |  |  |  |
| --- | --- | --- | --- | --- |
| int broad.core.primer3.Primer3PickingSummary.getNumSeqQuality | ( |  | ) |  |

---

The documentation for this interface was generated from the following file:

- java/broad/core/primer3/Primer3PickingSummary.java


---

Generated on Fri Aug 15 2014 15:10:55 for ESAT by  

 1.8.7
